# Adolescents as agents of healthful change through scientific literacy development: A school-university partnership program in New Zealand

**DOI:** 10.1186/s40594-017-0077-0

**Published:** 2017-09-06

**Authors:** Jacquie L. Bay, Mark H. Vickers, Helen A. Mora, Deborah M. Sloboda, Susan M. Morton

**Affiliations:** 1 Liggins Institute and Gravida, National Centre for Growth and Development, University of Auckland, Auckland, New Zealand; 2 Departments of Biochemistry and Biomedical Sciences, Obstetrics and Gynecology, and Paediatrics, Farncombe Family Digestive Health Research Institute, McMaster University, Hamilton, Canada; 3 Centre for Longitudinal Research-He Ara ki Mua, University of Auckland, Auckland, New Zealand; 4 Liggins Institute, University of Auckland, Private Bag 92109, Victoria Street West, Auckland, 1142 New Zealand

## Abstract

**Background:**

Scientific literacy development is widely emphasized as the overarching goal of science education. It encompasses development of understanding of the nature of science as well as knowledge, attitudes, and values that contribute to empowering adolescents to engage with and make evidence-based decisions about socioscientific issues. Scientific literacy development is enhanced when learning is contextualized in exploration of socioscientific issues.

Noncommunicable diseases (NCDs) associated with a combination of obesity and adverse environmental exposures are examples of pressing health-related SSIs facing the world today. Evidence emerging from the field of Developmental Origins of Health and Disease (DOHaD) has identified adolescence as a key life-phase where population-wide education-based interventions that empower teens to engage in science-based health-promoting behaviors could significantly change the course of this epidemic. To achieve this, learning resources that support scientific and health literacy development contextualized in issues linking NCD risk and DOHaD are required.

The Healthy Start to Life Education for Adolescents Project is a school-university partnership program designed to support scientific and health literacy development, knowledge translation, and participant-led actions relating to NCD risk prevention. This study assesses the impact of program participation in a cohort of 11–14-year-olds in New Zealand. Evaluation comprised analysis of individually matched questionnaires, pre-, 3 months, and 12 months post-intervention (*n* = 201) and 6 months post-intervention interviews (*n* = 40).

**Results:**

Positive engagement in science learning occurred. Positive changes in health-related awareness and attitudes 3 months post-intervention were sustained to 12 months. Adolescents reporting pre-intervention dietary behaviors associated with increased obesity risk reported sustained positive behavior changes (*p* < .001). Qualitative evidence revealed that these changes resulted from application of scientific and health literacy. This has the potential to improve long-term health outcomes for adolescents and their future offspring. Furthermore, feedback from parents demonstrated that adolescents became science communicators within their families.

**Conclusions:**

We demonstrated that contextualized learning promoting scientific and health literacy development facilitated knowledge translation. This allowed adolescents to decide if, and how, to use scientific evidence in relation to their current and future wellbeing. Exploration of the transferability of scientific and health literacy capabilities, and impacts on future health would enhance understanding of the value of the intervention.

**Electronic supplementary material:**

The online version of this article (doi:10.1186/s40594-017-0077-0) contains supplementary material, which is available to authorized users.

## Background

The development of scientific literacy is a key goal of science education (Hodson 2011), contributing to the capabilities required for engaged citizenship. Scientific literacy enables the use of science knowledge in decision-making relating to everyday occurrences as well as complex open-ended socioscientific issues. Understanding of the epistemology of science as a way of knowing, (the nature of science), is central to scientific literacy. However, knowledge and understanding of relevant scientific concepts is also required, alongside competencies associated with critical thinking, problem solving, communicating, acting autonomously (Rychen and Salganik 2003), and attending to moral and ethical ramifications (Sadler et al. 2004).

Development of scientific literacy and associated competencies is enhanced when learning is contextualized in real-world issues (Hipkins et al. 2014). The noncommunicable disease (NCD) epidemic is one such issue. Considered one of the most pressing socioscientific issues of our time, NCDs are responsible for considerable and growing social and economic burden (Bloom et al. 2011). Dominated by overweight, obesity, cardiovascular disease, cancers, and type 2 diabetes mellitus, these slow developing chronic conditions account for 64% of deaths globally. The burden of long-term morbidity and premature death associated with NCDs is disproportionately high in populations with limited resources (WHO 2015). NCD risk is impacted by a matrix of biological and socioecological factors that create rich contexts for learning associated with the development of scientific literacy (Bay et al. 2016). Because the NCD epidemic is a health-related socioscientific issue learning, using this context to facilitate scientific literacy development should also support health literacy development (Grace and Bay 2011). Similar to scientific literacy, health literacy enables the use of evidence in health-related decision-making (Nutbeam 2008).

Increasing understanding of the complexity of overweight, obesity, and NCD vulnerability has led to calls for multi-sectoral approaches to NCD risk reduction (Chestnov et al. 2013). These include prevention strategies prior to the onset of risk, known as primary prevention. Such strategies are informed by evidence from the field of Developmental Origins of Health and Disease (DOHaD) demonstrating that early life exposures, even before birth, as well as health status and nutritional exposures of either parent prior to conception, influence the vulnerability of an individual to later life obesity and NCDs (Hanson and Gluckman 2014).

Adolescence, a life stage where cognitive and lifestyle behaviors that track into adulthood are established, (Craigie et al. 2011; Steinberg 2005) offers significant opportunity for primary NCD risk prevention for the adolescent and their potential future offspring (Todd et al. 2015). Overweight and obesity in adolescence is known to persist into adulthood and impact future health (Alberga et al. 2012). Even if adolescence is significantly distanced from pregnancy, adolescent behaviors that track into adulthood will influence nutrition in the periconceptional period and during pregnancy, consequently influencing offspring vulnerability to obesity and related NCDs in later life (Bay et al. 2016). Thus, establishing positive nutritional and related lifestyle behaviors in the teenage years offers significant long-term health and social benefits for adolescents and their future offspring.

Life-long behaviors that develop during adolescence are influenced by educational, biological, cognitive, and socioecological factors. Therefore, the World Health Organization recognizes schools as having a key role in enabling primary NCD risk reduction (WHO 2016). However, school-based health interventions often have not been particularly successful (Khambalia et al. 2012) due to lack of connection to the core mission of schools (Waters et al. 2011). We have argued that this could be resolved by ensuring that school-based interventions are designed by educators working in partnership with health/science communities, integrate educational and health goals, utilize opportunities within existing curriculum objectives, are adaptable to enable differentiation, and involve educational as well as health-based evaluation (Bay et al. 2017; Bay and Vickers 2016). Science is a key learning area where opportunities for contextual learning supportive of scientific and health literacy development and primary NCD risk reduction exist. Such learning can offer adolescents the potential to apply scientific perspectives to decision-making that will influence their future NCD risk, as well as assisting them to understand the complexity of the NCD epidemic as a significant global issue.

New Zealand has a devolved curriculum centered on key competencies developed across all learning areas. In a devolved curriculum, high-level achievement aims grouped by strands are defined for each learning area. The responsibility of defining specific learning objectives and contexts within which learning will be set is devolved to the school. In science, the core strand, “Nature of Science,” is divided into four themes: understanding about science, investigating in science, communicating in science, and participating and contributing (bringing a scientific perspective to actions). Contextual strands (living-world, physical-world, material-world, and planet earth and beyond) support achievement aims defined in the Nature of Science strand alongside aims associated with understanding of scientific concepts (MoE 2007).

Representative of many settings, New Zealand is experiencing increasing overweight and obesity in children and adolescents as well as in adults (MOH 2012; Rajput et al. 2015; Utter et al. 2015). Considered together with information on the decreasing age of onset of type 2 diabetes mellitus in youth (Jefferies et al. 2012) and the increasing rates of pre-diabetes and diabetes in adults (Coppell et al. 2013), these data signal a growing future NCD burden for New Zealand. Thus, the NCD epidemic offers a highly relevant learning context for schools.

**Fig. 1:**
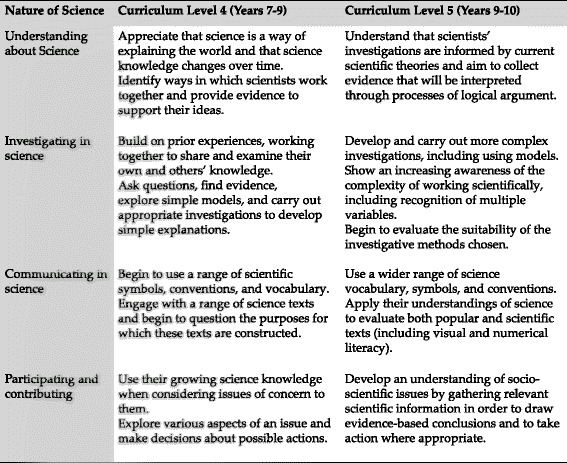
New Zealand Curriculum, Nature of Science Learning Objectives, levels 4 and 5 (MoE 2007)

The Healthy Start to Life Education for Adolescents Project (HSLEAP) is a multi-sectoral Science Education Partnership involving the Liggins Institute and schools (“LENScience Healthy Start to Life,” n.d.). Based on our “science for health literacy” pedagogical model (Grace and Bay 2011) programs facilitate learning that supports the development of capabilities associated with scientific and health literacies through exploration of aspects of the NCD epidemic. Programs are based on a narrative pedagogy, meaning that learning is facilitated by the exploration of stories associated with the socioscientific issue being considered. These stories include evidence from the community, health, and science. They enable adolescents to examine the relevance of the issue to their community, and the potential of primary NCD risk reduction to support improved long-term health and wellbeing. Programs support Nature of Science learning objectives at levels 4 and 5 of the New Zealand Curriculum, Fig. 1, ensuring validity for schools.

This study aimed to assess the potential of HSLEAP programs undertaken in year 7–10 (age 11–14 years) science classrooms in New Zealand to contribute towards development of science and health literacies, and simultaneously empower adolescents to engage in evidence-based decision-making in relation to lifestyle factors associated with nutrition. We have shown previously that exposure of year 7–10 students in New Zealand to HSLEAP programs stimulated evidence-based decision-making related to health-promoting behaviors at 3 months post-intervention (Bay et al. 2012a). This paper reports on educational and health-related impacts of this program exposure 12 months post-intervention.

## Methods

The study utilized a mixed methods approach within an individually matched repeated time-series design (Biglan et al. 2000), Fig. 2, approved by the University of Auckland Human Participants Ethics Committee (ref. 2009/426).

**Fig. 2:**
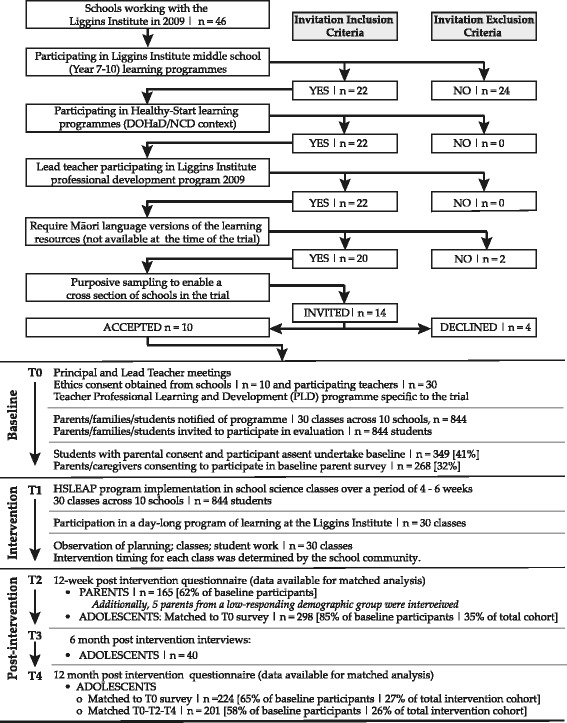
Study flow diagram

Analyzing individual change is important within community-based interventions as impacts will vary dependent on participant circumstances (Biglan et al. 2000). To achieve this, student questionnaire data was collected at baseline (pre-intervention) (T0), 3 months, (T2) and 12 months post-intervention (T4) and matched for each individual to assess the potential for program participation to support scientific and health literacy development, and for this to impact health and science-related knowledge, attitudes, and behaviors. Parent questionnaire data was collected and matched at T0 and T2. Matching of individual participant data enabled reporting of cohort-wide knowledge, attitude, and behavior trends at time-points, and change patterns based on aggregation of individual differences between time-points. Use of an explanatory mixed methods design (Punch 2009) enabled examination of the potential of qualitative data to corroborate quantitative data (Bryman 2008) and contributed evidence relating to why change did or did not occur.

### Context

The study was conducted in 30 classrooms ranging from years 7–10 across 10 Auckland schools. In New Zealand, curriculum levels span year levels. Curriculum levels 4 and 5 span years 7–10. Teachers design learning programs that enable students to progress through appropriate curriculum levels in mixed-ability classrooms. Hence, the diversity of year levels and schools in the study is appropriate and contributes towards addressing issues related to the impact of heterogeneity and complexity in school settings. Participating schools elected when to undertake the study to ensure integration into their science learning programs. The resulting data collection period spanned from 2010 to 2013.

### Recruitment

Schools were selected from a group of 46 participating in the Liggins Institute’s school partnership programs in 2009 (Bay et al. 2012b ). Inclusion criteria described in Fig. 2 generated a sample of 20 schools. Using purposive sampling, 13 schools were invited, aiming to create a sample representative of schools in the region. Prior to formal consent being sought, meetings occurred between invited schools and the Institute to discuss the study, intervention tool development, and research methods and to confirm appropriate recruitment processes. Nine schools accepted the invitation to participate, three of which were single-sex girls’ schools. On completion of the pre-intervention data collection a combination of two single-sex girls’ schools with high rates of participation and three co-educational schools from the lowest socioeconomic setting (SES) with low rates of participation had created a significant gender imbalance. To address this, a single-sex boys’ school was added to the study. Published evidence at 6 months post-intervention included nine schools (Bay et al. 2012a). Impacts to 12 months post-intervention reported here are from all ten schools.

Consent/assent to participate was obtained from principals, teachers, parents, and students. Meetings were held with all involved teachers and classes prior to written information being provided to families. Depending on school policy and practice, information about the study was also provided to families via school newsletters or parent meetings. Irrespective of participation in evaluation, all students were exposed to HSLEAP learning modules. Learning resources and teacher professional development were provided for each school.

### Intervention tools

**Fig. 3:**
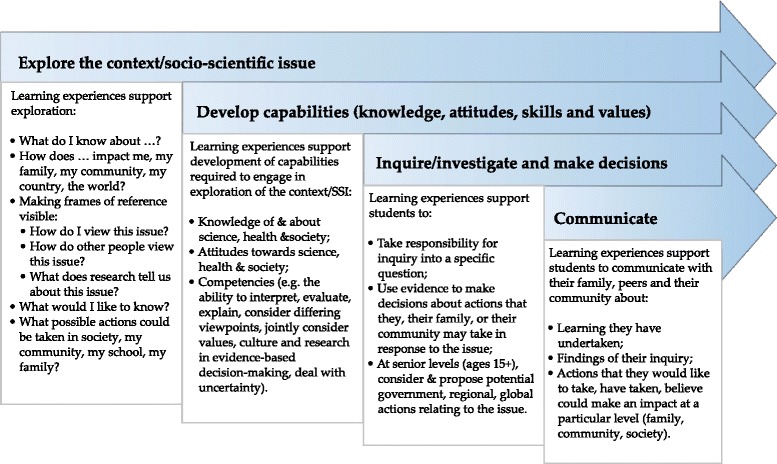
HSLEAP Learning and Teaching Framework

Intervention tools were developed by a multi-sectoral team led by science educators working as “intermediaries” (Bolstad and Bull 2013) capable of crossing science, education, and health. Tools consisted of adaptable learning modules based on the HSLEAP learning and teaching framework (Fig. 3) and contextualized in exploration of aspects of the NCD epidemic (Bay and Mora 2009a; Bay and Mora 2009b). The contexts were (a) nutrition (including early life) and later life obesity and cardiovascular disease vulnerability or (b) early life nutrition, early puberty and later life obesity, and NCD risk. Opportunities for students to explore scientific evidence are central to the framework. This is achieved via learning resources that use stories to enable students to explore the work of scientists and examine scientific evidence that is presented in formats that enable age-appropriate access (Bay et al 2012c). This enables students to traverse into the culture of science, encounter scientists, their stories, and scientific evidence and explore similarities and differences between scientists’ ideas and ways of thinking and their own. Combined with experiences of NCDs from families and/or communities, these narratives enable adolescents to construct and potentially act upon contextual understanding of evidence relating to life course approaches to NCD risk reduction. Each school developed a 4–6 weeks learning module (12–18 classroom hours) based on the framework and appropriate for their setting. During the module classes were exposed to “LENScience Face-to-Face,” a 1-day hands-on learning program exploring DOHaD research evidence at the Liggins Institute (“LENScience Face-to-Face,” n.d.).

### Data collection

Data sources included questionnaires, interviews, observations, and review meetings. Questionnaires for students, enabling quantitative analysis of knowledge, attitudes, and behaviors, utilized Likert attitude scales and closed items, and for parents in addition included open items (Additional file 1). Paper-based student questionnaires were completed in class under the guidance of a teacher who ensured students understood the nature of the questions and could seek clarification. Each school determined the format of the parent questionnaire. This was paper-based in nine schools and online via an email invitation in one school. Student interviews were conducted within the school environment by science educators within the research team who were not teaching the students (Additional file 1). Semi-structured open-ended questions were used. As reported previously (Bay et al. 2012a), difficulty in recruiting parents from schools in a low-socioeconomic demographic was addressed via interviews with five parents from this demographic. These semi-structured interviews, conducted by science educators from the research team, drew on the questions asked in the parent questionnaire. Review meetings with teachers and classroom observations confirmed that delivery of the intervention module was representative of the HSLEAP framework and sought feedback on student responses and potential improvements to the program.

### Data analysis

Publically available school demographic data (MoE 2012) provided information on the range of communities represented within the study. Quantitative data was analyzed using SPSS, (IBM Corp 2015). Descriptive statistics were used to identify frequencies. Comparison of response frequencies between groups was evaluated with Chi-square, Fisher’s exact and Mann-Whitney *U* tests as appropriate. Distribution of responses of self-matched ordinal data at pre-intervention. 3-, and 12 months post-intervention was analyzed using the Friedman test followed by post hoc assessment using the Wilcoxon Signed-Rank test. Matched binomial data were analyzed using Cochran’s *Q* test. Ordinal logistic regression was used to ascertain the effects of gender on cohort-wide response patterns. Bonferroni-Holm’s correction for multiple comparisons was applied (Aickin and Gensler 1996). Interviews were transcribed and anonymized prior to theme sorting using a constant comparative approach and inductive coding (Boyatzis 1998) in line with the relativist ontological and subjective epistemological approach (Levers 2013). Coding was conducted independently by two researchers and checked for inter-observer variability. Qualitative responses from parent questionnaires were treated similarly.

### Participants

Table 1 describes demographic characteristics and response rates for students. The students who participated in evaluation (*n* = 349) may not be representative of the intervention cohort (*n* = 844). Without access to school records, this could not be assessed. However, comparison of baseline data for those who did or did not complete all questionnaires demonstrated that the students who completed all questionnaires had a similar demographic and baseline response profile to those who responded to T0 and/or T0–T2 only Additional file 2. Where differences were found, the T0–T2–T4 matched group demonstrate attitudes and behaviors that are slightly less health-aware than the T0 and/or T0–T2 group. Parental evidence was received from 32% of the cohort at baseline with matched T0–T2 data available from 165 parents (Fig. 2), more than 50% of whom were linked to schools in the highest SES category.

**Table 1:** Cohort characteristics

		Schools in Auckland region^a^		Intervention participation and invitation to participate in evaluation						Matched pre- and post-intervention responses (students)										
										T0^b^		T0–T2 matched		% T0 retention	T0–T4 matched		% T0 retention	T0–T2-T4 matched		% T0 retention
				School		Classes		Students												
School community SES^c^	Decile 1–4	39	(33.9)	4	(40)	11	(36.7)	298	(35.3)	58	(19.5)	44	(14.8)	(75.9)	49	(21.7)	(84.5)	40	(19.9)	(69.0)
	Decile 5–7	20	(17.4)	1	(10)	6	(20.0)	174	(20.6)	111	(63.8)	99	(33.2)	(89.2)	68	(30.1)	(61.3)	64	(31.8)	(57.7)
	Decile 8–10	56	(48.7)	5	(50)	13	(43.3)	372	(44.1)	180	(48.8)	155	(52.0)	(86.1)	109	(48.2)	(60.6)	97	(48.3)	(53.9)
Gender	Male							290	(34.4)	123	(38.0)	106	(35.6)	(86.2)	87	(38.5)	(70.7)	78	(38.8)	(63.4)
	Female							554	(65.6)	226	(36.6)	192	(64.4)	(85.0)	139	(61.5)	(61.5)	123	(61.2)	(54.4)
School year level	Year 7–8							227	(26.9)	140	(61.7)	133	(44.6)	(95.0)	85	(37.6)	(60.7)	82	(40.8)	(58.6)
	Year 9–10							617	(73.1)	209	(33.9)	165	(55.4)	(78.9)	141	(62.4)	(67.5)	119	(59.2)	(56.9)
Median age at intervention										13y1m		12y11m			13y1m			13y0m		
Inter-quartile range										12y2m–14y1m		12y1m–14y0m			12y3m–14y1m			12y1m–14y0m		
Ethnicity (multiple responses accepted)	Māori									45	(12.9)	35	(11.7)	(77.8)	30	(13.3)	(66.7)	25	(12.4)	(55.6)
	Pacific									47	(13.5)	40	(13.4)	(85.1)	35	(15.5)	(74.5)	31	(15.4)	(66.0)
	Asian									50	(14.3)	40	(13.4)	(80.0)	34	(15.0)	(68.0)	26	(12.9)	(52.0)
	Indian									14	(4.0)	12	(4.0)	(85.7)	11	(4.9)	(78.6)	9	(4.5)	(64.3)
	NZ European									222	(63.6)	197	(66.1)	(88.7)	139	(61.5)	(62.6)	129	(64.2)	(58.1)
	Other									36	(10.3)	31	(10.4)	(86.1)	17	(7.5)	(47.2)	15	(7.5)	(41.7)
	Total	115		10		30		844		349	(41.4)	298	(35.3)	(85.4)	226	(26.8)	(64.8)	201	(23.8)	(57.6)

Values are numbers; (percentage by column), (percentage by row)

^a^Data from New Zealand Ministry of Education (Ministry of Education 2012), based on schools in the Auckland region comprising years 7–13, 9–13, 7–10, or 11–13

^b^Parents were encouraged to make the consent decision in consultation with their children. Where parents gave consent for children to participate, 96% of adolescents gave assent

^c^SES categorization for New Zealand schools is based on the SES of families within the school’s catchment area, calculated from census data relating to household income, educational qualifications, and occupation of adults within the household, household crowding, and income support provided to the household. Decile 10 includes the 10% of schools with the lowest proportion of low SES families within the catchment. Decile 1 includes the 10% of schools with the highest proportion of low SES families within the catchment (Ministry of Education, 2017)

The schools, nine of which were described in detail in Bay et al. 2012a, represented a cross section of communities from low to high SES. For New Zealand schools, this categorization is based on the SES of families within a school’s catchment area, calculated from census data relating to household income, educational qualifications, and occupation of adults within the household, household crowding, and income support provided to the household. Decile 10 includes the 10% of schools with the lowest proportion of low SES families within the catchment. Decile 1 includes the 10% of schools with the highest proportion of low SES families within the catchment (Ministry of Education 2017). We reported in Bay et al. 2012a that parents from schools in the lowest SES category (decile 1–4) were least likely to consent to students participating in the study. Discussion with teachers and parents suggested that this was associated with the requirement for written consent. Evaluation retention of students from this group was 1.2-fold greater than overall retention rates Table 1. However, at only 20% of the total cohort, this was inadequate to enable analysis of SES impacts on program response. Despite the female bias in the cohort noted earlier as a limitation of the study, T0–T2–T4 matched sample was 38% male, enabling limited evaluation of the impact of gender on program response.

## Results

If sustained actions are to emerge from program participation, it should be possible to identify behavioral and emotional engagement during and beyond the intervention period.

Teachers from diverse settings identified positive behavioral engagement during the intervention.

*“I have observed full engagement from students who often opt out.”* Teacher, decile 1–4

*“Students are seeing a different side of science. They are very positive about learning more about their health and wellbeing.”* Teacher, Decile 8-10

*“It got the students working and engaged.”* Teacher, Decile 5-7

Emotional engagement was indicated by factors such as enjoyment, interest, and identification of relevance of the program to personal situations.

*“It is all relevant. That enables the students to stay engaged.”* Teacher, Decile 8-10

*“I am interested that they appear so receptive. Great to see the kids’ enthusiasm in delivering their findings and information at the conference”* Parent, Decile 8-10, T2 survey

“*All the time he is talking about it. Before [the project] most of the time he didn’t talk about school but since this project started he has really started talking about this – about Health, Science and PE. He has been going on the computer and he has done a lot of research. To be honest I am amazed he has stepped out of the school space and is doing work on his own at home. He is motivated.”* Parent, Decile 1-4; T2 interview

Further examples of emotional engagement were reported previously in Bay et al. 2012a. When behavioral and emotional engagement leads to cognitive engagement, the potential exists for development and application of capabilities resultant in action-taking. To assess this, we looked at evidence relating to attitudes and understanding, followed by evidence associated with actions emerging from cognitive engagement.

### Awareness of and engagement with science

Assessment of understanding of key elements of the nature of science associated with the development of scientific literacy is presented in Tables 2 and 3. Pre-intervention the cohort generally understood science as an activity that seeks to understand the natural world via observation and inquiry. Investigation of common misconceptions relating to nature of science understanding sought to understand whether these were evident pre-intervention, and if so, whether program participation was associated with altered understanding. The misconceptions investigated were lack of creativity in science and certainty of scientific knowledge (Lederman et al. 2013). At T0, 64.7% of adolescents associated creativity and imagination with scientists. While overall cohort change was not significant, of the 69 students who responded Disagree (D), Strongly Disagree (SD), or Don't Know (DK) at T0, 45% changed to a position of Agree (A) or Strongly Agree (SA) post-intervention and retained this to T4, *p* < .001. In contrast, only 18% of the 126 students who initially responded SA/A changed to an uncertain or negative position at T4.

**Table 2:**
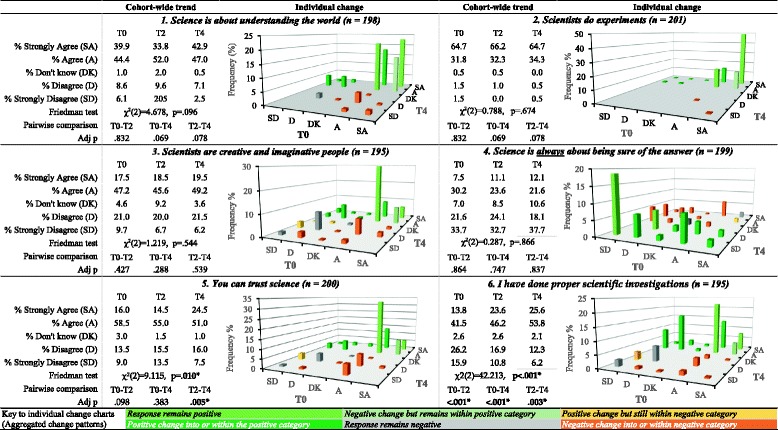
Perceptions of science: matched pre-post responses showing cohort-wide and individual change trends, *n* = 201

Variance in distribution of matched responses at T0, T2, and T4 was measured using the Friedman test. Post hoc pairwise comparisons were conducted using Wilcoxon Signed-Rank test

T0 pre-intervention, T2 6–12 weeks post-intervention, T4 12 months post-intervention, n number, Adj p adjusted significance values and include Bonferroni-Holm’s correction for multiple comparisons

*Bold: significant (*α* = 0.05)

**Table 3:** Perceptions of science: odds ratio (95% CI), male compared to female responses

Statement	Time	OR (male cf. female)	95% CI	χ^2^(1)	*p*
1. Science is about understanding the world	T0	0.8	0.5–1.4	0.493	.483
	T2	1.7	1.0–2.9	3.601	.058
	T4	1.3	0.8–2.3	0.901	.342
2. Scientists do experiments	T0	1.3	0.7–2.4	0.817	.366
	T2	1.4	0.7–2.5	1.061	.303
	T4	0.7	0.4–1.3	1.106	.293
3. Scientists are creative and imaginative people	T0	1.9	1.1–3.3	5.872	.015*
	T2	1.7	1.0–2.9	4.133	.042*
	T4	2.4	1.4–4.2	9.947	.002*
4. Science is always about being sure of the answer	T0	2.6	1.6–4.5	12.896	< .001*
	T2	3.0	1.7–5.0	16.258	< .001*
	T4	2.0	1.2–3.4	6.966	.008*
5. You can trust science	T0	1.4	0.8–2.5	1.571	.210
	T2	2.8	1.6–4.9	12.013	.001*
	T4	2.0	1.2–3.5	6.165	.013*
6. I have done proper scientific investigation	T0	1.4	0.8–2.4	1.519	.218
	T2	1.0	0.6–1.8	0.025	.875
	T4	1.8	1.0–3.1	4.102	.043*

The effect of gender on responses was measured using ordinal logistic regression with proportional odds

*T0* pre-intervention, *T2* 6–12 weeks post-intervention, *T4* 12 months post-intervention

*Significant *(α* = 0.05)

At T0, over 50% of students disagreed with the statement “*Science is always about being sure of the answer.”* Overall proportions of students taking this view did not alter significantly. However, 43% of the 75 students who pre-intervention responded SA/A moved to SD/D while only 22% of the 110 students who pre-intervention responded SD/D moved to SA/A. The odds of boys associating science with certainty was 2.6 times that of girls at T0, *p* < .001. At T2, this rose to 2.9 times that of girls, *p* < .001; while at T4, it reduced to 2.0 times that of girls, *p* = .008. These data should be of interest in boys’ schools as they suggest that unless teachers actively provoke discussions, it may be less likely that this frame of reference will be challenged.

People are unlikely to engage with and use scientific knowledge in decision-making if they do not trust science. Public distrust of science is common. It is associated with the use of science in regulatory capacities (Engdahl and Lidskog 2014) and risk analysis (Retzbach et al. 2016). Asked if science could be trusted, 74% of students responded SA/A at T0, with a small significant shift towards SA at T4. Of the 149 students responding SA/A at T0, only 18% moved to a negative response by T4 whereas 57% of the 51 students responding DK/D/SD at T0 moved to SA/A at T4. No significant gender difference was seen at T0. By T2, the odds of boys agreeing that science could be trusted was 2.8 times that of girls, *p* = .001, dropping to 2.0 at T4, *p* = .013. The relatively high level of trust in science exhibited may be supportive of engagement in exploration of NCD risk. The gender difference post-intervention warrants further investigation. Combined with the gender difference regarding the tentative nature of evidence this should be a point of discussion for teacher planning.

Doing science is associated with contributing towards nature of science understanding when combined with explicit instruction and opportunities for reflection (Kahana and Tal 2014). At baseline, 13.8%/41.5% of students respond SA/A to the statement “*I have done proper scientific investigations at school”* At T2, this shifted to 23.6%/46.2%, rising slightly at T4, 25.5%/53.8%, *p* < .001. Contributors to this shift could include changes in understanding of the process of science, and thus, appreciation of prior experiences, changes in teaching practice reflected in student experience, or a combination of both. It is likely that most students had undertaken open-ended investigations prior to the intervention as this is a component of the New Zealand Curriculum. During the intervention, all classes experienced learning exploring the process of science and carried out some form of open investigation. This suggests that the use of science narratives within the intervention may increase understanding of what science is and allow students to recognize science more readily.

**Fig. 4:**
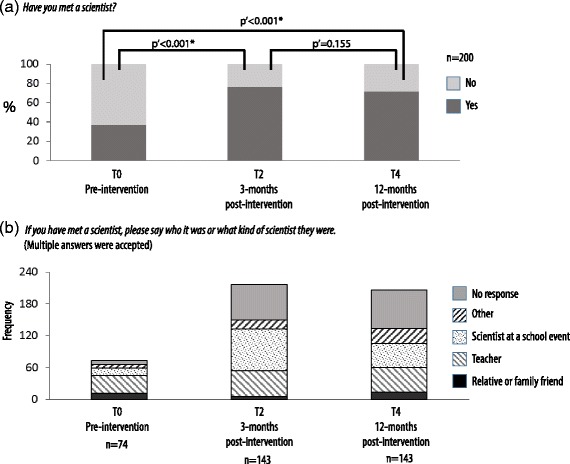
Experiences of meeting scientists. Matched pre-post responses, *n* = 201. **a** Matched pre-post responses to statement 7. *Have you met a scientist?* Variance in distribution of matched responses at T0, T2, and T4 was measured using the Cochran’s *Q* test, *Q* = 98.255, *p* < 0.001. Post hoc pairwise comparisons shown on the figure were conducted using McNemar’s test. The Bonferroni-Holm’s correction for multiple comparisons was applied. Binomial logistic regression demonstrated no significant difference between the responses of boys and girls. **b** Matched pre-post responses to statement 8. *If you have met a scientist, please say who it was or what kind of scientist they were?* Variance in the frequency of responses in each category was measured using the Chi-Squared test, χ^2^(8) = 53.48, * *p* **< .**001

Meeting scientists (actually and through narratives) enables students to explore epistemologies that may be different to their own. This supports the potential for frames of reference to be adapted to include evidence-based scientific perspectives in addition to perspectives arising from personal contexts. The majority of adolescents had little or no contact with scientists outside of their school environment Fig. 4. Pre-intervention, 37% of students identify with having met a scientist. Of this group, 63.5% identify either a teacher or a person they have met via a school event. Students did not identify all teachers as scientists. For example, in a school with six participating teachers, 39% of students identified having met a scientist at T0 and 68% of these qualified their answer by naming one or other of two teachers, both of whom had prior work experience in science. Post-intervention, the proportion of students identifying as having met scientists increases to greater than 70%, *p* < .001. While school-related encounters still dominate, there is a significant shift towards school events linked to the program. No significant differences were observed between the responses of boys and girls.

### Perceptions of the importance of health and lifestyle

**Fig. 5:**
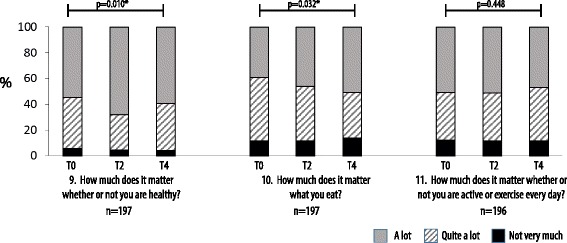
The importance of health and lifestyle. Matched pre-post responses, *n* = 201. Variance in proportion of responses confirming “a lot” vs less than “a lot” was assessed via related samples Cochran’s *Q* test, *significant (*α*=0.05). The effect of gender on responses was measured using a cumulative odds ordinal logistic regression with proportional odds. A significant difference in response based on gender was identified for statement 10 at T2 where the odds of boys identifying that what you eat mattered a lot was 0.4 times that of girls, *p* = 0.009

A comparison of matched pre-post responses to statements exploring perceptions of the importance of health and lifestyle was used to assess the impact of program participation on engagement with health as an issue of relevance to adolescents. Qualitative data at 6 months post-intervention demonstrated that students considered health as a concept that encapsulated physical, social, and emotional factors, and correctly interpreted concepts of healthy and unhealthy foods (Bay et al. 2012a). Pre-intervention students rated highly (“quite a lot” or “a lot”) the importance of “being healthy” (94.5%), “what you eat” (88.5%) and “daily exercise” (87.9%) Fig. 5. At baseline, students were less likely to rate “what you eat” as mattering “a lot” (39.0%) compared to being healthy (55.1%) and exercising daily (51.0%), assessed via Cochran’s *Q* test to be significant χ^2^(2) = 18.143, *p* < .001. Post hoc analysis revealed that the difference was between “what you eat” vs “being healthy,” *p* < .001, and “what you eat” vs “daily exercise,” *p* = .006. At 3 and 12 months post-intervention, there was a significant increase in students identifying “being healthy” χ^2^(2) = 9.297, *p* = .010 and “what you eat” as mattering “a lot” χ^2^(2) = 6.889, *p* = .032, suggesting that engagement was associated with positive attitudinal change. Conversely, there was no change in students’ perceptions of the importance of daily physical activity, a concept not explored within the program.

### Awareness of associations between nutrition and health

Responses to statements exploring awareness of associations between nutrition in early life and adolescence and health assessed the potential for the narrative-based exploration of DOHaD evidence within the learning program to support development of awareness of this evidence as a component of NCD vulnerability (Tables 4 and 5).

**Table 4:**
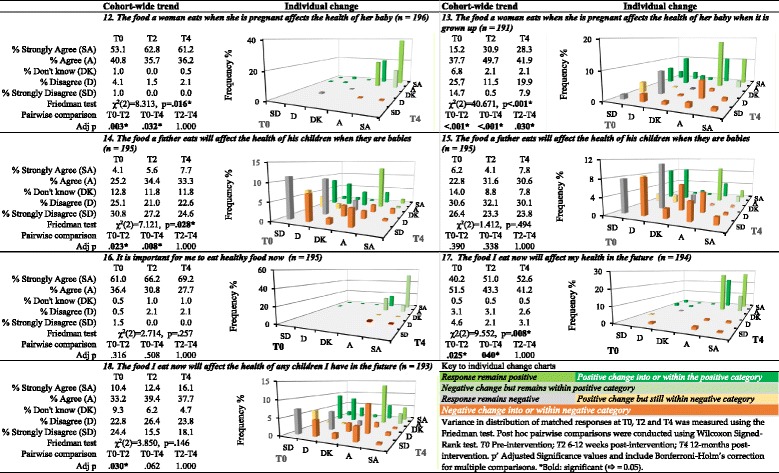
Awareness of associations between nutrition and health across the life course, *n* = 201

**Table 5:** Odds ratio (95% CI), awareness of associations between nutrition and health across the life course male compared to female responses

Statement	Time	OR (male cf. female)	95% CI	χ^2^(1)	*p*
12.The food a woman eats when she is pregnant affects the health of her baby	T0	0.6	0.4–1.4	2.908	.088
	T2	0.7	0.4–1.3	1.421	.699
	T4	0.6	0.4–1.1	2.421	.629
13. The food a woman eats when she is pregnant affects the health of her baby when it is grown up	T0	2.2	1.3–3.8	8.834	.003*
	T2	1.3	0.8–2.3	1.139	.286
	T4	2.2	1.3–3.8	8.417	.004*
14. The food a father eats will affect the health of his children when they are babies	T0	3.0	1.8–5.2	16.865	< .001*
	T2	2.2	1.3–3.7	8.626	.003*
	T4	2.9	1.7–4.9	14.907	< .001*
15. The food a father eats will affect the health of his children when they grow up	T0	2.6	1.6–4.5	12.988	< .001*
	T2	1.8	1.1–3.1	5.174	.023*
	T4	2.7	1.6–4.7	13.797	< .001*
16. It is important for me to eat healthy food now	T0	0.5	0.3–0.8	6.463	.011*
	T2	0.9	0.5–1.7	0.061	.805
	T4	0.5	0.3–0.9	4.699	.030*
17. The food I eat now will affect my health in the future	T0	0.8	0.4–1.3	0.924	.337
	T2	0.7	0.4–1.2	1.835	.176
	T4	1.1	0.6–1.9	0.061	.805
18. The food I eat now will affect my health of any children I have in the future	T0	2.1	1.2–3.5	7.550	.006*
	T2	1.4	0.8–2.2	1.517	.218
	T4	1.8	1.1–3.1	5.091	.024*

The effect of gender on responses was measured using ordinal logistic regression with proportional odds

*T0* pre-intervention, *T2* 6–12 weeks post-intervention, *T4* 12 months post-intervention

*Significant (*α* = 0.05)

Pre-intervention SA/A responses to statement 12 *“The food a woman eats when she is pregnant affects the health of her baby”* were high (53.1/40.8%). Significant positive shift towards SA occurred at T2 (62.8/35.7%) and was sustained to T4, (61.2%/36.2%), suggesting that the program increased awareness of this concept, *p* = .016. Gender difference in T0 response indicating that boys were less likely than girls to respond positively (*p* = .088) did not alter markedly post-intervention.

The concept of association between the nutritional environment of the mother during pregnancy and later life health (statement 13) elicited a wide range of pre-intervention responses. The odds of boys responding positively at T0 was 2.2 times that for girls *p* = .003. Strong positive change in awareness was observed at T2 and sustained to T4, *p* < .001. Of the 47.2% of participants who pre-intervention did not demonstrate awareness of this concept, 73.3% demonstrated awareness at 12 weeks post-intervention, 81.8% of whom continued to demonstrate this awareness at 12 months post-intervention. Awareness in girls increased markedly between T0 and T2, with the odds of boys responding positively at T2 lowering to 1.3 compared to that of girls *p* = .286. Retention of SA/A response was higher in boys (92%) compared to girls (83%) at T4. Correspondingly, the odds of boys responding positively at T4 increased to 2.2 that of girls, *p* = .004.

The learning resources did not explore associations between paternal nutritional environment prior to conception and offspring health. However, this concept was attracting increasing attention within the DOHaD community at the time of the intervention (Ng et al. 2010). It was raised as a question in teacher professional development, as well as by students in the one-day Liggins Institute program in the years prior to this intervention. Therefore, the concept was included as a point of emerging interest without exploration of evidence in the one-day Liggins Institute sessions attended by all participants. A small change in awareness was detected in responses to statement 14, suggesting that information sharing without examination of evidence has limited impact. When compared to the significant positive change in awareness of associations between maternal nutritional environment and later life offspring health, this confirms that for students to develop understanding of research evidence, they need to explore data rather than be told of findings.

Most adolescents receive signals from home, school, and community indicating the importance of healthy eating. Therefore, unsurprisingly 61.0/36.4% of students responded SA/A to statement 16 exploring the importance of eating healthy food. While not statistically significant, the change upwards to 69.2%/27.7% at 12 months post-intervention is promising. Gender-based differences were statistically significant at T0 and T4. At T0, the odds of boys responding positively to this statement were 0.467 that of girls, *p* = .011. Responses of boys shifted markedly at T2, bringing responses in line with those of girls. Girls responses at SA/A level continued to move upwards between T0 (69%/28.4%) and T4 (75.2%/22.2%). However, boys dropped back to 59.5%/36.5% at T4, still somewhat higher that their T0 rates of 50.0%/47.3%. Thus, the odds of boys responding positively at T4 lowered back to 0.507 that of girls, *p* = .030.

Positive (SA/A) responses to statement 17 “*The food I eat now will affect my health in the future*” were lower than those for statement 16 at T0, 40.2/51.5%. Post-intervention responses shifted significantly upwards and were sustained at 52.6/41.2% 12 months, *p* = .008.

Statement 18 “*The food I eat now will affect the health of any children I have in the future*” tested the potential for students to engage with DOHaD concepts in relation to their potential future offspring. Given their age (11–14 years), this was conceptually challenging as it related to events in their adult future. Responses at T0 were mixed, potentially reflecting this challenge. While overall, there was a positive shift in responses, significant moves occurred in both directions. This suggests that applying these concepts to their future as adults is challenging and should be explored further.

### Cognitive engagement and actions

Recognition of relevance, when followed by action signals investment in learning and is indicative of emotional and cognitive engagement, e.g.,

*“I had thought about it but I had never really done healthy eating before. Now I pay more attention to healthy eating and exercise. In P.E. I participate more. Like I used to just stand there but now I take part and it is really fun. [Before] I was ashamed, but now I enjoy it and I really take part and I eat healthy too. I eat vegetables.”* Year 9 female, decile 1–4, 6 months post-intervention

The potential of program participation to facilitate nutritional behavior change was assessed using matched self-reported evidence relating to eight key food categories Tables 6 and 7. Not all adolescents need to make nutritional behavior changes. Therefore, we divided the assessment of behavior change into two categories based on the risk-level represented by baseline patterns. We reported at 12 weeks post-intervention indicators of positive change in students whose pre-intervention nutritional behaviors could increase later risk of overweight/obesity and NCD. This was triangulated with interview evidence indicating that change was determined by adolescents in response to evidence they had explored in the HSLEAP programs (Bay et al. 2012a). Matched analysis demonstrates statistically significant sustained change in self-reported behaviors from T0 to T2 and T4 for all food categories for students in the “at risk” group Table 6. Consistent with our reported 12 weeks post-intervention evidence, some negative behavior changes were observed at 12 months post-intervention in the group for whom pre-intervention behavior was not in the “at risk” category. However, the odds of positive behavior change for students in the “at risk” group were significantly higher than the odds of students in the “low/no risk” group making negative changes Table 7.

**Table 6:** Change in self-reported diet behaviors indicated by individually matched pre- and post-intervention responses, *n* = 167*

Food item	Self-reported consumption pattern defined as indicating risk	T0 responses in at risk category			T0–T2–T4		Pre- to 12 weeks post-intervention (T0–T2)			Pre- to 12 months post-intervention (T0–T4)			Odds_male_/Odds_female_ T0 to T4 positive change
		*n*	%	Odds_male_/odds_female_ Being in the at risk category at T0	χ^2^(2)	*p*	Positive change (%)	Negative change (%)	*p*′	Positive change (%)	Negative change (%)	*p*′	
Potato chips (crisps)	> once per week	69	*42.3*	1.4 (95% CI 0.7 to 2.5), χ^2^(1) = 0.878, *p* = .349	30.516	**<** .001*	*39.1*	*1.5*	< .001*	*42.9*	*5.7*	< .001*	0.6 (95% CI 0.2 to 1.5), χ2(1) = 1.142, *p* = .285
Fried food (e.g., hot chips, fried chicken, burgers)	≥ once per week	93	*58.5*	1.3 (95% CI 0.7 to 2.4), χ^2^(1) = 0.606, *p* = **.**436	17.857	< .001*	*43.0*	*16.1*	< .001*	*36.6*	*9.7*	< .001*	0.8 (95% CI 0.4 to 1.9), χ2(1) = 0.171, *p* = .679
Soft drinks (fizzy, cordials, sports drinks)	≥ 2–4 times per week	41	*24.5*	2.1 (95% CI 1.0 to 4.3), χ^2^(1) = 4.129, *p* = .042*	13.904	< .001*	*36.6*	*7.3*	< .01*	*43.9*	*7.3*	< .01*	1.5 (95% CI 0.4 to 4.8), χ2(1) = 0.384, *p* = .535
Sweet snacks (e.g., biscuit, muesli bar, sweet (candy))	> 2-4 times per week	28	*17.8*	1.0 (95% CI 0.5 to 2.1), χ^2^(1) = 0.009, *p* = **.**925	18.764	< .001*	*42.9*	*N/A*	.001	*50.0*	*N/A*	< .001*	1.2 (95% CI 0.6 TO 2.4), χ2(1) = 0270, *p* = .604
Green vegetables (e.g., spinach, beans, lettuce)	< Daily	58	*36.5*	1.2 (95% CI 0.6 to 2.3), χ^2^(1) = 0.336, *p* = **.**540	20.520	< .001*	*46.6*	*5.2*	< .001*	*39.7*	*10.3*	< .01*	1.2 (95% CI 0.4 to 3.3), χ2(1) = .137, *p* = .711
Starchy vegetables (e.g., sweet potato, potato, pumpkin)	≤ once per week	26	*16.4*	1.5 (95% CI 0.6 to 3.5), χ^2^(1) = 0.874 6, *p* = **.**350	26.275	< .001*	*73.1*	*0.0*	< .001*	*73.1*	*3.8*	< .001*	2.9 (95% CI 0.4 to 18.9), χ2(1) = 1.271 *p* = .260
Fruit (e.g., apples, pears, bananas)	< Daily	57	*35.0*	1.9 (95% CI 1.0 to 3.6), χ^2^(1) = 3.545 6, *p* = **.**060	16.528	< .001*	*38.6*	*5.3*	< .001*	*45.6*	*14.0*	< .01*	3.0 (95% CI 1.0 to 8.3), χ2(1) = 4.255 *p* = .039*
Raw fruits and vegetables	< Daily	88	*54.3*	1.1 (95% CI 0.6 to 2.1), χ^2^(1) = 0.159 6, *p* = .690	23.452	< .001*	*48.9*	*11.4*	< .001*	*52.3*	*19.3*	.002*	0.8 (95% CI 0.4 to 1.8), χ2(1) = 0.230 *p* = .632

The Friedman test was used to measure variance in distribution at T0, T2, and T4. Post hoc pairwise comparisons were conducted using Wilcoxon Signed-Rank test or related-samples sign test

*T0* pre-intervention, *T2* 6–12 weeks post-intervention, *T4* 12 months post-intervention, *n* number, p*significant (*α* = 0.05). p' adjusted significance values and include Bonferroni-Holm’s correction for multiple comparisons (Aickin, M. and Gensler, H. 1996)

*An administrative error in data collection at one site reduced the number of valid responses to food frequency questions at T0. Hence, *n* = 167 rather than 201

**Table 7:** Comparative behavior change patterns (at risk vs no/low risk), *n* = 167

		Pre-intervention to 12 months post-intervention behavior change								
		T0 behavior category = risk					T0 behavior category = no/low risk			
Food item	Self-reported consumption pattern defined as indicating risk	*n*	% Negative change towards greater risk	% No change	% Positive change towards no/low-risk category	% Positive change into the no/low-risk category	*n*	% Retains position in no/low-risk category	% Negative change into at risk category	Odds ratio T0-risk/T0-no/low-risk T0–T4 change towards opposite category
Potato chips (crisps)	> once/week	69	*5.8*	*52.2*	*7.2*	*34.8*	94	*80.9*	*19.1*	3.1 (95% CI 1.2 to 6.2), χ^2^(1) = 10.15, *p* < .001*
Fried food (e.g., hot chips, fried chicken, burgers)	≥ once per week	93	*9.7*	*53.8*	*17.2*	*19.4*	66	*66.7*	*33.3*	1.2 (95% CI 0.6 to 2.2), χ^2^(1) = 0.18, *p* = .671
Soft drinks (fizzy, cordials, sports drinks)	≥ 2–4 times per week	41	*7.3*	*48.8*	*4.9*	*39.0*	123	*87.8*	*12.2*	5.6 (95% CI 2.5 to 12.8), χ^2^(1) = 19.23, *p* < .001*
Sweet snacks (e.g., biscuit, muesli bar, sweet (candy))	> 2–4 times per week	28	*N/A*	*50.0*	*N/A*	*50.0*	131	*82.4*	*17.6*	4.696 (95% CI 1.974 to 11.173), χ^2^(1) = 13.60, *p* < .001*
Green vegetables (e.g., spinach, beans, lettuce)	< Daily	58	*10.3*	*50.0*	*5.2*	*34.5*	101	*84.2*	*15.8*	3.5 (95% CI 1.7 to 7.4), χ^2^(1) = 11.29, *p* = .001*
Starchy vegetables (e.g., sweet potato, potato, pumpkin)	≤ once per week	26	*3.8*	*23.1*	*7.7*	*65.4*	133	*91.0*	*9.0*	27.3 (95% CI 9.6 to 78.2), χ^2^(1) = 56.85, *p* < .001*
Fruit (e.g., apples, pears, bananas)	< Daily	57	*14.0*	*40.4*	*17.5*	*28.1*	109	*84.9*	*15.1*	5.7 (95% CI 2.7 to 12.0), χ^2^(1) = 56.85, *p* < .001*
Raw fruits and vegetables	< Daily	88	*19.3*	*28.4*	*29.5*	*22.7*	74	*73.0*	*27.0*	3.0 (95% CI 1.5 to 5.7), χ^2^(1) = 10.61, *p* = .001*

*T0* pre-intervention, *T2* 6–12 weeks post-intervention, *T4* 12 months post-intervention, *n* number, p*significant (*α* = 0.05). The effect of T0 response group (“at risk” vs “low/no risk”) on change response was measured using ordinal logistic regression with proportional odds

### Cognitive engagement and communication

At T2, increased parental awareness of DOHaD-related concepts was identified (Bay et al. 2012a) and 73% of parents responding to a request to comment on the program (*n* = 55) indicated interest in learning about aspects of science explored within the program. Additionally, 37 of the 40 adolescents interviewed at 6 months post-intervention talked about engaging their family in learning from the program and 19 discussed application of their learning in evidence-based actions, examples of which we reported previously (Bay et al. 2012a). These data indicate that students became science communicators in their families. This is a sign of cognitive engagement. In addition to supporting family level behavior change reported in Bay et al. 2012a, this has supported parents to develop an understanding of science as a human endeavor of relevance in their community.

“Back in my day science at school was not so relevant to the children’s world. Soana* is telling me about what she is doing. She is really interested and they are looking at how things work. That has changed my mind on what science is so I can see there are branches of it in everyday things and I can see the value of science for her. When I came to the parent’s night I was interested and wanting to know more about what Soana* was learning at school and I went away learning something, and I enjoyed the evening. I wanted to come to this meeting because she has been talking about science ever since this program started and she has said that she likes science so I thought well I should come and support her.” Parent, decile 1–4 school; 6 months post-intervention, *name altered

This type of impact is valued by schools as it promotes the worth of science education within communities.

*“The value of the program for parents in our community is that they have begun to see what science is a little more. The students are taking back ideas to their families and their communities. We are getting a lot more understanding about how science is impacting the community. We are getting more students wanting to take science – they can see the relevance of it. There is a strong link to their lives.”* HOD science, decile 1–4 school

## Discussion

This study tested the potential of narrative-based science learning contextualized in NCD-related issues to support scientific and health literacy development and facilitate health-promoting actions. Participation was strongly associated with engagement, known to be a prerequisite to the development of capabilities associated with critical citizenship, and associated with higher levels of scientific literacy (Caygill and Sok 2008). Increased understanding of the culture, nature, and process of science as well as research evidence associated with life course understanding of NCD risk was observed. Greater knowledge change was associated with exposure to learning resources enabling students to explore evidence compared to questions relating to evidence not presented in this manner. Assessment of nutritional behavior change demonstrated that the positive change in the at risk group was significantly higher than negative change in the “no/low-risk” group for all but one food item (fried foods). Combined with the significant qualitative evidence from the 6 months post-intervention interview data demonstrating that the observed positive behavior changes were associated with application of evidence-based thinking (Bay et al. 2012a), these data indicate that students in the at risk group made and sustained evidence-based decisions in relation to learning associated with the intervention. The high level of communication into the family by students is encouraging as it supports evidence linked to the importance of family engagement in achieving positive adolescent health promotion (Todd et al. 2015).

In some aspects of the evaluation, we identified small increases in understanding and capability development between 3 and 12 months post-intervention, indicative of ongoing development beyond the intervention period. This reflects the notion that each learning module in a program contributes to overarching educative goals. Therefore, ideas explored will be linked into learning beyond the module, supporting ongoing capability development.

The impacts of this study sit in contrast to the questionable value of school-based health interventions expressed in the literature (Khambalia et al. 2012). Unusually for school-based health interventions, experienced science educators embedded in a health-research setting led the intervention design. This enabled a program supportive of educational as well as health/science goals (Bay  et al. 2016) and set within a core curriculum area (WHO 2016), addressing issues that connect to the core mission of schools (Waters et al. 2011). Ensuring that the intervention resources supported teachers to develop locally relevant programs addressed issues associated with the impact of school diversity on intervention impacts (Keshavarz Mohammadi et al. 2010).

Evaluation recognized that for education to impact health, assessment must look at whether learning took place before asking whether this learning impacted health. Ideally, the program should also support measurement of long-term health impacts, an issue that we hope to address in the future.

Exploration of health issues in classrooms should be approached with appropriate consideration for pastoral care. The adaptable learning modules used were designed in collaboration with stakeholder schools following 4 years of trials where the context was explored in the Institute classroom with over 5000 students. Care was taken to ensure that negative messages were not presented. While the potential exists for exploration of health-related issues to have negative impacts, and some argue that it is dangerous (Fitzpatrick and Tinning 2014), the overwhelming feedback we received was positive. Anecdotal evidence from teachers during the pre-study developmental period identified that rather than promoting negative responses, the programs created opportunities for positive classroom discussions that challenged individual blame responses to NCD-related health issues that are reflected in social norms and can promote bullying in classrooms.

### Limitations

The study is limited by several factors, including lack of matched control schools. However, strong arguments exist demonstrating the limitation of control studies in complex educational settings (Berliner 2002; Maxwell 2004; Sadler et al. 2016). Factors within the study design addressed internal validity. Individually matched analysis was used throughout the study, addressing issues of individual variance and fixed confounding variables. The use of 30 classrooms across 10 schools contributed to addressing factors associated with heterogeneity in school settings. Biglan et al. argue that time-series studies should be considered as experimental rather than quasi-experimental (Biglan et al. 2000). However, in this study, we were limited to a single pre-intervention baseline where to be considered experimental, the baseline questionnaires should be repeated to determine stability prior to intervention (Biglan et al. 2000). When conducting research in schools, the use of curriculum time for evaluation must be carefully considered. Most New Zealand schools schedule 3 hours per week for science learning within the timetable in years 7–10. The evaluation processes required 5 hours of this time. The addition of a further hour to collect a second baseline was rejected by participating schools. This was addressed by comparing baseline evidence with known evidence relating to nature of science understanding and nutritional behaviors in New Zealand adolescents. The high level of nature of science understanding at baseline was reflective of international assessment of nature of science understanding in New Zealand 15-year-olds (MoE 2009). Baseline food frequency data was comparative to patterns in the New Zealand National Youth Nutrition Survey (Clinical Trials Research Unit Synovate 2010). Awareness of associations between nutrition in early life and later life health was consistently low across all 10 schools at baseline, measured over a period of 2 years as each school timed the study to suit their own needs. This is parallel to data we have from 900 adults in the Auckland region (Bay et al. 2015), indicating reliability of the baseline. Furthermore, change data was triangulated with interview data from parents and students. Collectively, this indicates that it is highly likely that changes found in individually matched pre-post assessments can be attributed to intervention participation.

Generalizability of findings is likely to be context dependent and associated with opportunity for nature of science exploration within national curricula. The model is designed to be adapted by teachers to fit within the socioecological context of their community and classroom. However, the impact of teacher quality, experience, preparation, and beliefs are known to be significant factors influencing learning outcomes for students (Darling-Hammond et al. 2009; Hattie 2012). We have not assessed the impact of teachers on learning outcomes for students. This would be a significant limitation if the study had it been conducted in only one school with few teachers and used set rather than adaptable learning programs. However, the data represents evidence from 30 classrooms in 10 schools where teachers used professional judgment to adapt the program to the context of their community. Further investigation examining outcome variation associated with teacher experience and context would be valuable.

As noted, the study cohort presented limitations in terms of the potential to explore the impact of gender, age, and SES. Gender differences observed should be treated cautiously as we were not able to assess differences in SES and age due to the mid-SES group being from single-sex girls’ schools and the year 7–8 cohort being from mid- and high-decile schools only. Further research is underway to address these questions.

## Conclusions

The World Health Organization is promoting the importance of curriculum-embedded school-based partnerships to support primary NCD risk reduction. This study examined whether addressing known shortcomings relating to school-based health promotion, such as shared vision, and understanding of pedagogy and practice could demonstrate the value of combining educative and health goals in support of DOHaD translation in the adolescent life stage. Our study suggests that the use of a narrative-based pedagogy centered on development of scientific literacy is effective in promoting evidence-based actions by adolescents that are supportive of long-term health. Utilization of mixed methods enabled student, teacher, and parent voices to identify associations between observed knowledge, attitude, and behavior changes and program participation. Strong links to the goals of the national curriculum combined with evidence of positive change in nature of science understanding validated the use of science learning time and offers strong potential for sustainability and future development. Further development and testing of tools and contexts associated with primary NCD risk reduction linked to learning objectives in science, as well as other core learning areas should be explored. Understanding of impacts associated with teacher diversity, student age, gender, and socioecological setting are required, along with assessment of relative sustainability and contextual transferability of scientific literacy capabilities developed in this manner.

## Additional files

Additional file 1: Questionnaires and semi-structured interviews (Student and Parent). This file contains questions used to collect the data presented in the paper. (DOCX 35 kb)

Additional file 2: Comparison of T0 responses from participants who did not and who did provide T2 and T4 responses. This file contains data comparing the baseline evidence from the group of students who completed all three questionnaires with the group who only completed some questionnaires. (DOCX 26 kb)

## Abbreviations

DOHaD: Developmental Origins of Health and Disease
HSLEAP: Healthy Start to Life Education for Adolescents Project
NCD: Noncommunicable diseases
SES: Socioeconomic status
T0: Time 0: baseline
T1: Time 1: intervention
T2: Time 2: 3 months post-intervention
T3: Time 3: 6 months post-intervention
T4: Time 4: 12 months post-intervention
