# Supplementary material for: Adolescents as agents of healthful change through scientific literacy development: A school-university partnership program in New Zealand
Source: Int J STEM Educ. 2017 Sep 6;4(1):15. doi: 10.1186/s40594-017-0077-0 (PMC6310384; doi:10.1186/s40594-017-0077-0)
Supplement: Supplementary file 1 — Questionnaires and semi-structured interviews (Student and Parent). This file contains questions used to collect the data presented in the paper. (DOCX 35 kb) [file 40594_2017_77_MOESM1_ESM.docx]

Healthy Start to Life: Adolescent Education Programme

Student Questionnaire

Thank you for participating in this questionnaire. We are looking forward to working with you and your class.

Section One: About You.

| *Name* |  | | | | |
| --- | --- | --- | --- | --- | --- |
| *Date of Birth* |  | | | | |
| *School* |  | | | | |
| *Year Level* | Year 7 🞎 | *Year 8* 🞎 | *Year 9* 🞎 | *Year 10* 🞎 | *Year 11* 🞎 |

| Ethnicity – Tick all the boxes that represent you | |  |  |  |
| --- | --- | --- | --- | --- |
| Māori |  |  | I am Female |  |
| NZ Pakeha / NZ European |  |  |  |  |
| Pasifika |  |  |  |  |
| Asian |  |  | I am  Male |  |
| Indian |  |  |  |  |
| Other |  |  |  |  |

Section Two: About Science

|  | **Yes** | **No** |
| --- | --- | --- |
| Have you ever met a scientist? |  |  |
| If your answer was yes, can you tell me who it was or what kind of scientist they were? |  | |

| For each of these statements, tick one box to tell me what you think...... | Strongly Agree | Agree | Disagree | Strongly Disagree | I don’t know |
| --- | --- | --- | --- | --- | --- |
| I enjoy doing science at school |  |  |  |  |  |
| I am good at science |  |  |  |  |  |
| You can trust science |  |  |  |  |  |
| Scientists do experiments |  |  |  |  |  |
| Scientists do work that is important |  |  |  |  |  |
| Science is about understanding the world |  |  |  |  |  |
| Science is **always** about being sure of the answer |  |  |  |  |  |
| Scientists need to be good at English |  |  |  |  |  |
| Scientists need to be good at Maths |  |  |  |  |  |
| I have done proper scientific investigations |  |  |  |  |  |
| Scientists are creative & imaginative people |  |  |  |  |  |
| When I am an adult, I would like to have a job that involves science..... |  |  |  |  |  |

Section Three: About Health

|  | Very Good | Good | OK | Bad | Very Bad |
| --- | --- | --- | --- | --- | --- |
| My personal health is..... |  |  |  |  |  |

| On a school day I usually get up at _______________________ o’clock in the morning |
| --- |
| On a school day I usually go to sleep at ____________________ o’clock at night |

|  | Very healthy | Healthy | OK | Unhealthy | Very Unhealthy |
| --- | --- | --- | --- | --- | --- |
| My lifestyle is usually ... |  |  |  |  |  |
| The food I eat is usually... |  |  |  |  |  |

|  | A lot | Quite a lot | Not very much | Not at all |
| --- | --- | --- | --- | --- |
| How much does it matter what you eat? |  |  |  |  |
| How much does it matter whether or not you are active or exercise every day? |  |  |  |  |
| How much does it matter whether or not you are healthy? |  |  |  |  |
| How often do you get to choose what you eat? |  |  |  |  |

|  | My parents or grandparents | My brothers and sisters | My Friends | None of these people |
| --- | --- | --- | --- | --- |
| If you do not choose what you get to eat, who does? |  |  |  |  |

|  | Breakfast | Lunch | Dinner | Snacks |
| --- | --- | --- | --- | --- |
| If you do get to choose what you eat, which meals do you choose? |  |  |  |  |

| When I choose my food the **most important** thing is: | What my friends think | Good taste | Healthy food | Quick to get it |
| --- | --- | --- | --- | --- |
|  |  |  |  |  |

| For each of these statements, tick one box to tell me what you think...... | Strongly Agree | Agree | Disagree | Strongly Disagree | I don’t know |
| --- | --- | --- | --- | --- | --- |
| The food a woman eats when she is pregnant affects the health of her baby |  |  |  |  |  |
| The food a father eats will affect the health of his children when they are babies |  |  |  |  |  |
| The food I eat now will affect my health in the future |  |  |  |  |  |
| The food I eat now will affect the health of any children I have in the future |  |  |  |  |  |
| The food a woman eats when she is pregnant affects the health of her baby when it is grown up |  |  |  |  |  |
| The food a father eats will affect the health of his children when they grow up |  |  |  |  |  |
| It is important for me to eat healthy food now |  |  |  |  |  |

| I eat or drink... | Everyday | 2-4 times a week | Once a week | Less than once a week | Never |
| --- | --- | --- | --- | --- | --- |
| Potato chips or crisps |  |  |  |  |  |
| Soft drinks  (e.g. fizzy drinks, cordial, sports drinks) |  |  |  |  |  |
| Sweet snacks (e.g. biscuits, muesli bars and sweets/lollies) |  |  |  |  |  |
| Green vegetables (e.g. spinach, beans, lettuce, peas) |  |  |  |  |  |
| Starchy vegetables ( e.g. kumara, potatoes, pumpkin, yams etc.) |  |  |  |  |  |
| Fruit (e.g. apples, pears, bananas) |  |  |  |  |  |
| Raw fruits and vegetables |  |  |  |  |  |
| Fried food (e.g. hot chips or fries, fried chicken, burgers) |  |  |  |  |  |

**Student Interview Questions**

1. What do you remember about the XXXXX (name relevant to each school) programme?
2. Did you learn anything during the programme? If yes – prompt question: Actions?
3. What did you enjoy most about the programme?
4. Did you talk to anyone about the programme?

Prompt questions: Who? What sort of things did you talk about?

Why did you want to talk to them about the programme?

1. How could the programme be improved?
2. What were the benefits of doing the programme for you?
3. Is being healthy important to you? Can you tell me why/why not?
4. Can you tell me about how the food we eat and our lifestyle affects our health and wellbeing?
5. What sorts of things influence the food that you choose for yourself?
6. Does diet and lifestyle during our teenage years affect our health and wellbeing?

Prompt questions: How? What sort of effects does it have?

1. Does a mother’s diet and lifestyle during pregnancy affect the health / wellbeing of the baby?

Prompt questions: How? When? What sort of effects does it have? What about Dad?

1. Have you ever met a scientist?

Prompt questions: Who / where? What did you think of that?

1. Do you think it is important to find out about what scientists are doing?

Prompt questions: Why / why not. Is it easy/hard to find out that kind of information?

1. If you had the opportunity, would you participate in something like this again? Why / why not?

Healthy Start to Life: Adolescent Education Programme

Parent/Caregiver Pre-Questionnaire

Thank you for participating in this questionnaire.

Section One: About You.

| *Name^[[1]](#footnote-1)^* |  | | | | |
| --- | --- | --- | --- | --- | --- |
| *Child’s name* |  | | | | |
| *Child’s School* |  | | | | |
| *Child’s Year Level* | Year 7 🞎 | *Year 8* 🞎 | *Year 9* 🞎 | *Year 10* 🞎 | *Year 10* 🞎 |

| Ethnicity – Tick all boxes that relate to you | |  |  |  |
| --- | --- | --- | --- | --- |
| Māori |  |  | Female |  |
| NZ Pakeha / NZ European |  |  |  |  |
| Pasifika |  |  |  |  |
| Asian |  |  | Male |  |
| Indian |  |  |  |  |
| Other |  |  |  |  |

Section Two: About Science

| For each of these statements, tick one box to tell me what you think...... | Strongly Agree | Agree | Disagree | Strongly Disagree | I don’t know |
| --- | --- | --- | --- | --- | --- |
| Science is an important subject for children at school |  |  |  |  |  |
| Scientists do work that is important for society |  |  |  |  |  |
| Scientists can be trusted |  |  |  |  |  |
| Science is always about being sure of the answer |  |  |  |  |  |
| Scientists are creative & imaginative people |  |  |  |  |  |
| I would be pleased if my child went on to become a scientist |  |  |  |  |  |
| I will actively encourage my child to continue studying science at school |  |  |  |  |  |
| If my child became a scientist they would easily get a good job |  |  |  |  |  |

Section Three: About Health

|  | A lot | Quite a lot | Not very much | Not at all |
| --- | --- | --- | --- | --- |
| How much does it matter what you and your family eat? |  |  |  |  |
| How much does it matter whether or not you and your family are active or exercise every day? |  |  |  |  |
| How much does it matter whether or not you and your family are healthy? |  |  |  |  |

| The food that my family eat is influenced by... | A lot | Quite a lot | Not very much | Not at all |
| --- | --- | --- | --- | --- |
| Convenience |  |  |  |  |
| How healthy it is |  |  |  |  |
| Cost |  |  |  |  |
| What the children will eat |  |  |  |  |
| What the children want to eat |  |  |  |  |
| What the adults want to eat |  |  |  |  |
| Taste |  |  |  |  |
| Cultural or family traditions |  |  |  |  |

| For each of these statements, tick one box to tell me what you think... | Strongly Agree | Agree | Disagree | Strongly Disagree | I don’t know |
| --- | --- | --- | --- | --- | --- |
| The food a woman eats when she is pregnant affects the health of her baby |  |  |  |  |  |
| The food a father eats prior to conception will affect the health of his children when they are babies |  |  |  |  |  |
| The food I eat now will affect my health in the future |  |  |  |  |  |
| The food a woman eats when she is pregnant affects the health of her baby when it is grown up |  |  |  |  |  |
| The food a father eats prior to conception will affect the health of his children when they grow up |  |  |  |  |  |
| It is important for me and my family to eat healthy food |  |  |  |  |  |

| For each of these statements, tick one box to tell me what you think... | Strongly Agree | Agree | Disagree | Strongly Disagree | I don’t know |
| --- | --- | --- | --- | --- | --- |
| My child is usually interested in the work they do in science at school |  |  |  |  |  |
| My child talks at home about what they do in science at school |  |  |  |  |  |
| As a family we learn from what the children do at school |  |  |  |  |  |

**The parent/caregiver *post-intervention* questionnaire included the following additional questions**

| For each of these statements, tick one box to tell me what you think...... | Strongly Agree | Agree | Disagree | Strongly Disagree | I don’t know |
| --- | --- | --- | --- | --- | --- |
| My child has been interested in the work they have been doing in science |  |  |  |  |  |
| My child has talked at home about what they have been doing in science |  |  |  |  |  |
| As a family we have learnt from what the children have been doing at school |  |  |  |  |  |

| Please comment on anything you have found interesting about the work that your child’s class have been doing at school in the Healthy Start to Life Programme |
| --- |
|  |

1. Note: The relationship between the adult and the child was identified via the consent form completed by the adult. [↑](#footnote-ref-1)
