# Supplementary material for: Adolescents as agents of healthful change through scientific literacy development: A school-university partnership program in New Zealand
Source: Int J STEM Educ. 2017 Sep 6;4(1):15. doi: 10.1186/s40594-017-0077-0 (PMC6310384; doi:10.1186/s40594-017-0077-0)
Supplement: Supplementary file 2 — Comparison of T0 responses from participants who did not and who did provide T2 and T4 responses. This file contains data comparing the baseline evidence from the group of students who completed all three questionnaires with the group who only completed some questionnaires. (DOCX 26 kb) [file 40594_2017_77_MOESM2_ESM.docx]

Comparison of T0 responses from participants who did not (T0 or T0-T2) and who did (T0-T2-T4 Matched) provide both T2 and T4 responses. Numbers are values (percentages).

Demographic factors

|  | Gender | | | | Decile Band | | | | | | Year Level | | | |
| --- | --- | --- | --- | --- | --- | --- | --- | --- | --- | --- | --- | --- | --- | --- |
| T0 or T0-T2 Response only | Male | | Female | | Decile 1-4 | | Decile 5-7 | | Decile 8-10 | | Year 7-8 | | Year 9-10 | |
|  | 45 | (30.4) | 103 | (69.6) | 18 | (12.2) | 47 | (31.8) | 83 | (56.1) | 58 | (39.2) | 90 | (60.8) |
| T0-T2-T4 matched | 77 | (38.8) | 124 | (61.7) | 40 | (19.9) | 64 | (31.8) | 97 | (48.3) | 82 | (40.8) | 119 | (59.2) |
|  | χ^2^=2.341, p=.126 | | | | χ^2^=4.083, p=.130 | | | | | | χ^2^=0.092, p=.762 | | | |

Attitudes to health and wellbeing

|  | How much does it matter  whether or not you are healthy | | How much does it matter  what you eat | | How much does it matter  whether or not you exercise or are physically active every day | |
| --- | --- | --- | --- | --- | --- | --- |
|  | T0/T0-T2 Only | T0-T2-T4 match | T0/T0-T2 Only | T0-T2-T4 match | T0/T0-T2 Only | T0-T2-T4 match |
| A lot | 102 (69.9) | 109 (55.1) | 66 (44.9) | 78 (39.0) | 65 (4.5) | 101 (51.0) |
| Quite a lot | 38 (26.0) | 78 (39.4) | 55 (37.4) | 99 (49.5) | 67 (5.9) | 73 (36.9) |
| Not Very Much | 4 (2.7) | 11 (5.6) | 24 (16.3) | 22 (11.0) | 11 (7.5) | 23 (11.6) |
| Not at all | 2 (1.4) | 0 (0.0) | 2 (1.4) | 1 (0.5) | 3 (2.1) | 1 (0.5) |
|  | U=16,599, z=2.701, p=**.007*** | | U=14,902, z= .239, p=.811 | | U=13,780, z=-.817, p=.414 | |

Awareness of associations between nutritional environment and health (self)

|  | The food I eat now will affect my health in the future | | | It is important for me to eat healthy food now | | |
| --- | --- | --- | --- | --- | --- | --- |
|  | T0/T0-T2 Only | T0-T2-T4 match | | T0/T0-T2 Only | T0-T2-T4 match | |
| Strongly Agree | 56 (38.1) | | 78 (39.8) | 91 (61.9) | | 120 (61.2) |
| Agree | 73 (49.7) | | 102 (52.0) | 48 (32.7) | | 71 (36.2) |
| Don’t know | 3 (2.0) | | 1 (0.5) | 1 (0.7) | | 1 (0.5) |
| Disagree | 10 (6.8) | | 6 (3.1) | 3 (2.0) | | 1 (0.5) |
| Strongly Disagree | 5 (3.4) | | 9 (4.6) | 4 (2.7) | | 3 (1.5) |
|  | U=13,872, z=-.654, p=.513 | | | U=14,341, z=-.084, p=.933 | | |

Awareness of associations between nutritional environment and health (intergenerational)

|  | The food a woman eats when she is pregnant affects the health of her baby | | | The food a woman eats when she is pregnant affects the health of her baby when it is grown up | | | The food I eat now will affect the health of any children I have in the future | | |
| --- | --- | --- | --- | --- | --- | --- | --- | --- | --- |
|  | T0/T0-T2 Only | T0-T2-T4 match | | T0/T0-T2 Only | T0-T2-T4 match | | T0/T0-T2 Only | T0-T2-T4 match | |
| Strongly Agree | 74 (50.7) | | 104 (52.8) | 21 (14.4) | | 29 (15.2) | 15 (10.3) | | 20 (10.3) |
| Agree | 58 (39.7) | | 81 (41.1) | 49 (33.6) | | 72 (37.7) | 40 (27.4) | | 64 (32.8) |
| Don’t know | 2 (1.4) | | 2 (1.0) | 10 (6.8) | | 13 (6.8) | 16 (11.0) | | 19 (9.7) |
| Disagree | 9 (6.2) | | 8 (4.0) | 41 (28.1) | | 49 (25.7) | 31 (21.2) | | 45 (23.1) |
| Strongly Disagree | 3 (2.1) | | 2 (1.0) | 25 (17.1) | | 28 (14.7) | 44 (30.1) | | 47 (24.4) |
|  | U=13,854, z=-.652, p=.515 | | | U=13,281, z=-.850, p=.396 | | | U=13,307, z=-1.062, p=.288 | | |

Nutritional behaviors

|  | Potato Chips | | Soft Drinks | | Fried Foods | |
| --- | --- | --- | --- | --- | --- | --- |
|  | T0/T0-T2 Only | T0-T2-T4 match | T0/T0-T2 Only | T0-T2-T4 match | T0/T0-T2 Only | T0-T2-T4 match |
| Daily | 12 (10.2) | 7 (4.1) | 6 (5.1) | 6 (3.6) | 2 (1.7) | 5 (3.0) |
| 2-4 Times/wk | 38 (32.2) | 63 (37.3) | 23 (19.5) | 35 (21.0) | 19 (16.1) | 32 (19.2) |
| Once per week | 24 (20.3) | 43 (25.4) | 27 (22.9) | 42 (25.1) | 36 (30.5) | 60 (35.9) |
| < Once per week | 38 (32.2) | 50 (29.6) | 49 (41.5) | 72 (43.1) | 56 (47.5) | 66 (39.5) |
| Never | 6 (5.1) | 6 (3.6) | 13 (11.0) | 12 (7.2) | 5 (4.2) | 4 (2.4) |
|  | U=10,001, z=.045, p=.964 | | U=, z=-.467, p=.641 | | U=13,307, z=-1.062, p=.288 | |
|  | Green Vegetables | | Fruit | | Raw Fruit and Vegetables | |
|  | T0/T0-T2 Only | T0-T2-T4 match | T0/T0-T2 Only | T0-T2-T4 match | T0/T0-T2 Only | T0-T2-T4 match |
| Daily | 88 (73.9) | 105 (63.3) | 90 (75.6) | 111 (66.1) | 52 (44.1) | 78 (46.4) |
| 2-4 Times/wk | 23 (19.3) | 53 (31.9) | 24 (20.2) | 44 (26.2) | 43 (36.4) | 43 (25.6) |
| Once per week | 5 (4.2) | 6 (3.6) | 3 (2.5) | 8 (4.8) | 15 (12.7) | 23 (13.7) |
| < Once per week | 2 (1.7) | 1 (0.6) | 2 (1.7) | 4 (2.4) | 6 (5.1) | 17 (10.1) |
| Never | 1 (0.8) | 1 (0.6) | 0 (0.0) | 1 (0.6) | 2 (1.7) | 7 (4.2) |
|  | U=10,810, z=1.661, p=.097 | | U=10,998, z=1.804, p=.071 | | U=10,286, z=.580, p=.562 | |

Variance in response patterns at baseline (T0) from the two groups were assessed using Fisher’s exact and Mann-whitney U tests. *Bold: significant (α=0.05). T0 = Pre-intervention; T2 = 6-12 weeks post-intervention; T4 = 12-months post-intervention; n = number;
